# ChannelsDB 2.0: a comprehensive database of protein tunnels and pores in AlphaFold era

**DOI:** 10.1093/nar/gkad1012

**Published:** 2023-11-13

**Authors:** Anna Špačková, Ondřej Vávra, Tomáš Raček, Václav Bazgier, David Sehnal, Jiří Damborský, Radka Svobodová, David Bednář, Karel Berka

**Affiliations:** Department of Physical Chemistry, Faculty of Science, Palacký University, tř. 17. listopadu 12, 771 46 Olomouc, Czech Republic; Loschmidt Laboratories, Department of Experimental Biology and RECETOX, Faculty of Science, Masaryk University, Kamenice 5, 625 00 Brno, Czech Republic; International Clinical Research Center, St. Anne's University Hospital Brno, Pekařská 53, 656 91 Brno, Czech Republic; CEITEC – Central European Institute of Technology, Masaryk University Brno, Kamenice 5, 625 00 Brno, Czech Republic; National Centre for Biomolecular Research, Faculty of Science, Masaryk University Brno, Kamenice 5, 625 00 Brno, Czech Republic; Department of Physical Chemistry, Faculty of Science, Palacký University, tř. 17. listopadu 12, 771 46 Olomouc, Czech Republic; CEITEC – Central European Institute of Technology, Masaryk University Brno, Kamenice 5, 625 00 Brno, Czech Republic; National Centre for Biomolecular Research, Faculty of Science, Masaryk University Brno, Kamenice 5, 625 00 Brno, Czech Republic; Loschmidt Laboratories, Department of Experimental Biology and RECETOX, Faculty of Science, Masaryk University, Kamenice 5, 625 00 Brno, Czech Republic; International Clinical Research Center, St. Anne's University Hospital Brno, Pekařská 53, 656 91 Brno, Czech Republic; CEITEC – Central European Institute of Technology, Masaryk University Brno, Kamenice 5, 625 00 Brno, Czech Republic; National Centre for Biomolecular Research, Faculty of Science, Masaryk University Brno, Kamenice 5, 625 00 Brno, Czech Republic; Loschmidt Laboratories, Department of Experimental Biology and RECETOX, Faculty of Science, Masaryk University, Kamenice 5, 625 00 Brno, Czech Republic; International Clinical Research Center, St. Anne's University Hospital Brno, Pekařská 53, 656 91 Brno, Czech Republic; Department of Physical Chemistry, Faculty of Science, Palacký University, tř. 17. listopadu 12, 771 46 Olomouc, Czech Republic

## Abstract

ChannelsDB 2.0 is an updated database providing structural information about the position, geometry and physicochemical properties of protein channels—tunnels and pores—within deposited biomacromolecular structures from PDB and AlphaFoldDB databases. The newly deposited information originated from several sources. Firstly, we included data calculated using a popular CAVER tool to complement the data obtained using original MOLE tool for detection and analysis of protein tunnels and pores. Secondly, we added tunnels starting from cofactors within the AlphaFill database to enlarge the scope of the database to protein models based on Uniprot. This has enlarged available channel annotations ∼4.6 times as of 1 September 2023. The database stores information about geometrical features, e.g. length and radius, and physico-chemical properties based on channel-lining amino acids. The stored data are interlinked with the available UniProt mutation annotation data. ChannelsDB 2.0 provides an excellent resource for deep analysis of the role of biomacromolecular tunnels and pores. The database is available free of charge: https://channelsdb2.biodata.ceitec.cz.

## Graphical Abstract

**Graphical Abstract:**
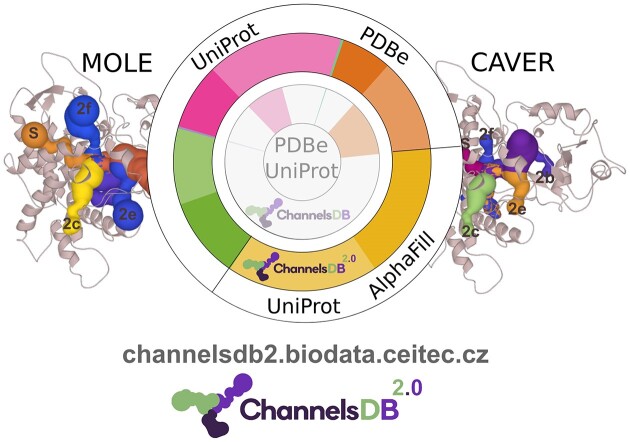

## Introduction

There are over 200 000 structures present in the Protein Data Bank (PDB) (1,2). Still, while the growth of the number of experimental structures is substantial, it was dwarfed by the availability of high-quality structural models due to AlphaFold (3). Over 200 000 000 structural models were collected in AlphaFoldDB (4), thus enriching our view of the protein structural universe, including the channels within proteins important for the access routes to active sites of soluble proteins (tunnels) or transport through transmembrane proteins (pores).

To analyse the access routes important for ligand transport from/into protein binding sites, our teams have previously developed highly used tools MOLEonline (5,6), CAVER (7) and Caver Web (8). These tools have enabled the detection and analysis of channels, tunnels, and pores for over a decade, and their results have been collected in the publicly accessible database ChannelsDB (9). Since 2018, the database has grown from ∼28 000 entries to ∼40 000 experimental entries due to the assignments of channels for newer structures, the addition of results on cognate ligands based on recent studies (10,11), and complementing MOLE channels with CAVER ones to get consensus prediction. Moreover, the data in ChannelsDB were based only on a limited number of experimental structures. Identifying the start of the tunnel is nontrivial to fill the knowledge gap in model Alphafold structures. Fortunately, the AlphaFill database (12) has been developed to fill the Alphafold models with ligands and cofactors that can serve as a starting point for channel analysis. AlphaFill provides another ∼25 000 model entries and is continuously growing.

ChannelsDB has been freely accessible since March 2017 at https://channelsdb.ncbr.muni.cz/ and last year had close to 5000 users/year. This frequent usage of ChannelsDB is supported by its integration into a comprehensive PDBe-KB database (13). The usage of ChannelsDB is also reinforced by the fact that its source services (MOLE, MOLEonline, CAVER and Caver Web) are popular tools for detecting protein channels, tunnels and pores in protein structures.

## Database content

**Figure 1.**
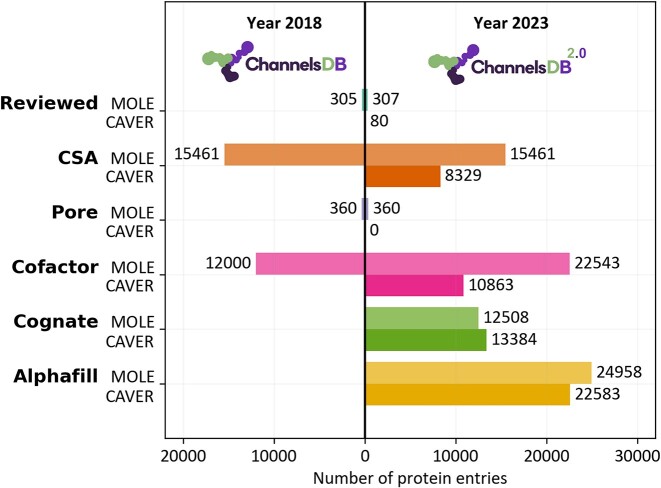
The growth of the content of ChannelsDB from the original version released in 2018 (9) to the current day (this study). The colour coding indicates different data categories - light coloured are MOLE calculated entries, and CAVER is a darker colour. The updated database ChannelDB 2.0 contains three times more data than the original release from 2018. The database has grown approximately ∼4.6 times in size of channel annotations. Calculation and visualisation of tunnels and channels using MOLE 2.0 is newly complemented by analysis using CAVER 3.02, thus allowing utilisation of consensus tunnel annotation.

ChannelsDB 2.0 is a comprehensive database that combines data layers obtained from PDBe (2) and UniProt (14), along with predicted protein structures obtained based on deep learning and stored in a database AlphaFill (12). Experimental structures from the PDB database and model structures from AlphaFill database can be accessed through separate search windows, enabling the linkage to the corresponding stored tunnels, pores, and channels. UniProt provides essential details about the studied proteins, including residue annotations. ChannelsDB 2.0 utilises several diverse methods to obtain tunnels and pores (Figure 1):

- ∼80 and ∼300 proteins with **manually reviewed** tunnels from literature calculated by CAVER 3.02 (7) and MOLE 2.0 (15), respectively. See details of the calculation below.
- ∼8300 and ∼15 500 proteins with tunnels starting from catalytic sites annotated by **Catalytic Site Atlas** (16) calculated by CAVER and MOLE, respectively.
- ∼350 proteins with **transmembrane pores** predicted by the pore mode of MOLEonline (5,6).
- ∼10 800 and ∼22 500 proteins with tunnels starting from **cofactors** calculated by CAVER and MOLE, respectively.
- ∼13 400 and ∼12 500 newly added proteins with tunnels containing **cognate ligands**, based on experimental evidence or functional annotations (10,11) calculated by CAVER and MOLE, respectively. More information is below in the next section.
- ∼22 600 and ∼25 000 newly added protein models with tunnels starting from cofactors transplanted into AlphaFold models via the **AlphaFill** (12) database, calculated by CAVER and MOLE, respectively. More information is below in the next sections.

### Cognate dataset

The Cognate dataset utilises data collected from publications by Tyzack *et al.* and Vavra *et al.* (10,11) about enzyme-ligand complexes ranked by the similarity of the bound ligand with the cognate ligand, a putative natural substrate or product of the enzyme, from the KEGG database (17), calculated by the PARITY algorithm (11). We used all of the enzymes whose bound ligands had a similarity score above 0.6 and successfully calculated tunnels in their crystal structures, resulting in 13531 CAVER and 12508 MOLE entries.

### AlphaFill dataset

As of 21 August 2023, the AlphaFill database (12) contained 2325 transplanted compounds, with a cumulative total of 337 060 structures, when considering a 70% identity threshold. The same 58 distinct cofactors were selected for tunnel calculation as in the cofactor dataset, leading to 33 689 structures, out of which 16 257 (48%) or 24 958 (74%) structures contained at least one tunnel calculated with CAVER or MOLE, respectively.

### Tunnels calculated by MOLE 2.0

Except transmembrane pore dataset calculation, where MOLEonline (5,6) pore mode is used by first identifying the membrane boundaries and then calculating transmembrane pore from one boundary to another through the protein centre; standard MOLE 2.0 calculation parameters (15) were used, including BottleneckRadius 1.2 Å (the size of a water molecule), BottleneckTolerance 3 Å (allowing for the length of a bottleneck), MaxTunnelSimilarity 0.7 (to discard too similar tunnels), OriginRadius 4.0 Å (to optimise starting point around specified geometric centre of selection), and SurfaceCoverRadius 10 Å (to distinguish endpoints by distance of at least 10 Å). In the case of the Cognate dataset, the starting point identified by the CAVER procedure below was used as an input for MOLE 2.0 calculation.

### Tunnels calculated by CAVER 3.02

In the first step of CAVER 3.02 calculations (7), the analysed structure is cleared off any molecules that are not cofactors based on the lists of cofactors from FPOCKET (18) and CoFactor Database (19). The next essential step is calculating pockets by FPOCKET 2 (18) with parameters: -m 2.8 -n 10 -r 4.5 -s 2.5. Then, the starting points for tunnel calculation by CAVER 3.02 are defined similarly to the previously mentioned pipeline (10), with slight differences depending on the data source:

- In the case of the Cognate dataset (see above), we select the pocket that contains the first location of the bound ligand molecule, find the nearest protein chain, and select the protein residue with the Cα atom closest to the geometrical centre of the chain. Next, the geometrical centre of the pocket is calculated, and in the previously selected residue, the nearest side-chain atom to the pocket centre is found. After that, we save the coordinates of the point placed further into the space by 0.5 Å on the vector connecting the closest atom and the centre of the pocket. The coordinates of this point are saved as the starting point for the CAVER 3.02 tunnel calculation.
- For the data from ChannelsDB (reviewed, CSA and cofactor datasets), each starting point is found in the pocket containing the original MOLE starting point for tunnels in the database.
- The structures from AlphaFill are cleared from all molecules but the matched cofactor. In this case, we analyse the pockets that contain the reactive atoms from each cofactor position in the model structures.

The tunnels are then calculated with the same settings for all structures by CAVER 3.02 with a probe radius of 0.9 Å and remaining default settings.

The conversion algorithm processes protein tunnel data from the CAVER results for visualisation. The ‘residues.txt’ file is used to identify the residues around each tunnel, and these are stored as HetResidues in a JSON file for subsequent visualisation on the ChannelsDB web page. Next, the algorithm uses the ‘summary.txt’ file to obtain the starting points of the tunnels. It then divides each analysed tunnel into layers based on the surrounding residues. Calculating the five nearest atoms to the centre axis of the tunnel determines the residues for each layer, with a maximum of five residues per layer. The algorithm calculates the MinRadius for each layer, representing the minimum radius within that layer. It also calculates the start and end layer radius for the visualisation. Tunnel properties are determined by analysing the properties of lining amino acids and summing their relevant characteristics, normalised by the number of amino acids considered. Finally, all calculated data, including HetResidues, layer information, and tunnel properties, are written to a JSON file for display on the ChannelsDB 2.0 web page.

The ChannelsDB 2.0 homepage allows users to perform queries for protein structures from PDBe or AlphaFill databases containing annotated channels. This flexible search functionality allows users to use any PDB-related metadata, such as PDB ID, protein name, protein family, cofactor, ligand, author or even the journal in which the research was published. The underlying search engine is powered by the PDBe RESTful API, which efficiently and accurately retrieves relevant records. The ChannelsDB 2.0 website offers comprehensive documentation explaining the methodology used for channel annotation within the database and several examples of typical channel systems.

## Results and discussion

### Case study 1—tunnels in AlphaFill and X-ray structures of cytochrome P450 2D6

**Figure 2.**
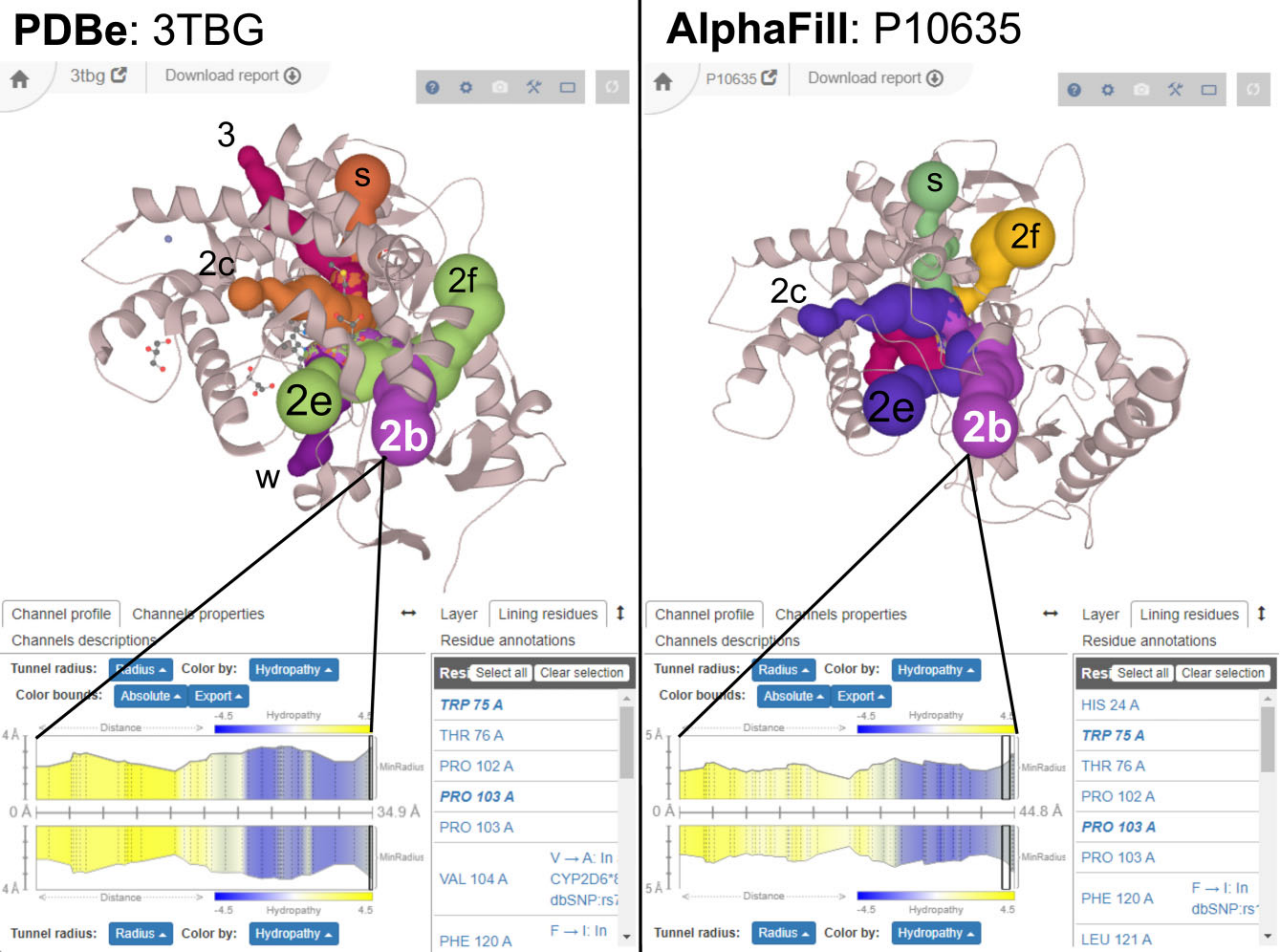
Comparison of named reviewed cytochrome P450 2D6 tunnels from X-ray structure (left, PDB ID 3TBG) from Wang *et al.* (20) with automatically calculated tunnels from AlphaFill model (right, UniProt ID P10635). The structure is oriented from the membrane perspective (see the position of the transmembrane anchor in the AlphaFill model pointing towards the viewer), and thus, the major substrate entry tunnels (2b, 2e and 2f) are the most visible and retained in the AlphaFill model. Also, major product release tunnels (2c and S) pointing above the membrane (and sidewise for the viewer) are retained. A narrower rare egress tunnel 3 and water channel (W) leading to the proximal side toward the cytosol are missing. As seen from the tunnel 2b profile at the bottom of the figure, the profiles and their properties are almost identical, including channel-lining residues (bottom right) and their UniProt annotation (e.g. Phe 120 in chain A). All ligands in both structures were hidden for clarity.

Cytochromes P450 (CYPs, EC 1.14.-.-) are a versatile and most typically monooxygenase enzyme family known in all kingdoms of life. In humans, these enzymes are involved in the metabolism of fatty acids, steroids or drug metabolism. One of the drug-metabolizing enzymes is CYP2D6, which is responsible for the metabolism and elimination of approximately 25% of commercially available drugs—especially those containing nitrogen (20). The active sites of oxidoreductases are typically buried (21); hence, CYPs have their heme-containing active sites accessible only via tunnels. Tunnels have nomenclature with respect to secondary structures (22). Membrane-bound channels are used for substrate entry, while the channels leading above the membrane are used for metabolite release (23). This fact is reflected by the properties of individual tunnels based on their lining residues (Figure 2).

CYP2D6 is thus a great, well-described system, which can be used to showcase the similarity of tunnels within AlphaFold models to the X-ray structure side-by-side (Figure 2). Since AlphaFold structures do not contain ligands nor cofactors, we have used transplanted cofactors from the AlphaFill database as starting points for tunnel calculation. As shown in Figure 2 from the membrane perspective, most of the named reviewed channels from the X-ray structure are also present in the AlphaFill model - especially the major access (2b, 2e, 2f) and egress (2c, S) channels. Narrower channels (3, W) are, however, not retained, and the imperfect transplantation of the heme cofactor opens an additional tunnel following the edges of the heme cavity. We can thus conclude that AlphaFold models can be, in principle, ‘tunnelled’ for ligand access/egress routes.

### Case study 2—tunnels calculated based on cognate ligands in haloalkane dehalogenase LinB

Haloalkane dehalogenases (EC 3.8.1.5) belong to the α/β-hydrolase superfamily of enzymes. They perform the catalysis of the carbon-halogen bond cleavage in halogenated compounds. Due to their robust properties and wide substrate range, these enzymes have been used in various practical applications, including biodegradation, bioremediation, and cell imaging (24). The access tunnels in haloalkane dehalogenases have been well studied and are a target of engineering by mutagenesis to change their properties (25,26).

**Figure 3.**
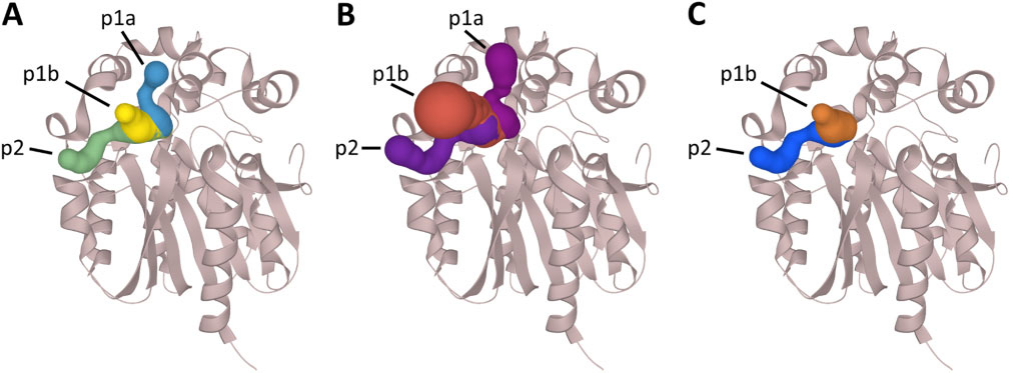
Visualisation of tunnels identified in the structure of haloalkane dehalogenase LinB (PDB ID: 2BFN). (**A**) Reviewed tunnels extracted from the publication by Brezovsky *et al.* (26), tunnels calculated based on starting points defined by an automatic pipeline using the information about bound and cognate ligands with (**B**) MOLE 2.0 and (**C**) CAVER 3.02. Even with the automatically generated starting points and default settings, both tools correctly identified the main tunnel p1 and the slot tunnel p2.

Haloalkane dehalogenase LinB from *Sphingobium japonicum* UT26 is involved in the biodegradation pathway to degrade the organochlorine pesticide lindane. LinB is one of the few dehalogenases capable of slowly processing the toxic environmental pollutant 1,2,3-trichloropropane (27). The crystal structure with bound product (2*S*)-2,3-dichloropropan-1-ol was obtained in a soaking experiment with 1,2,3-trichloropropane (PDB ID 2BFN) and was selected for this case study. This structure is a part of the new Cognate dataset. The bound ligand in the crystal structure was paired with the information about the cognate reaction and ligand of the enzyme. The location of the bound molecule was used to select the binding pocket automatically, define the starting point and analyse the tunnels. We have previously shown that the approach employing cognate ligands as a starting point has a higher success rate than selecting the pocket based on FPOCKET and Druggability scores (10). Both MOLE and CAVER found the main tunnels in the structure of the dehalogenase (Figure 3).

### Usage and integration with other databases and bioinformatics tools

The ChannelsDB database was accessed nearly 5000 times last year. ChannelsDB data is a part of the PDBe-KB database (12,13), integrating a rich set of annotations of biomacromolecules. ChannelsDB was also included in the PDBsum database (27), summarising key information about individual protein structures. Visualisation of protein tunnels and pores, developed for ChannelsDB, was included in a frequently used molecular viewer, LiteMol (26). ChannelsDB is also referenced in bio.tools (28)—a comprehensive database of bioinformatics services, and in FAIRsharing (29), a database of data and metadata standards interrelated to databases and data policies. The accessibility of ChannelsDB can also benefit from the inclusion of REST API service that can be found together with its documentation at the address https://channelsdb2.biodata.ceitec.cz/api.

### Limitations

Ligand transport through the protein is dynamic in nature, and a single snapshot, albeit experimental, might not be representative enough. Compared to a real system, some channels may be closed and some open. Hence, it is advisable to test multiple protein conformers or even run molecular dynamics to evaluate the influence of protein movement on the ligand transport process along the channel. Unfortunately, ChannelsDB 2.0 is based on static structures either from PDBe or AlphaFill databases. If multiple structures are available, we advise analysing the channels in all.

Another limitation is the automated database-wide configuration of both software tuned according to experience for their best performance for typical organic molecular ligands. For larger systems interacting with larger biomacromolecules (e.g. ribozymes), it is necessary to change parameters accordingly, especially the probe size. Any parameter changes can significantly affect the accuracy and calculated tunnels in protein structures. We are also limited to subsets of cofactors and cognate ligands for tunnel calculation. Unoccupied or unannotated active sites are thus missed, but they can be tested for channel presence with both software packages: https://mole.upol.cz/ or https://loschmidt.chemi.muni.cz/caverweb/.

Finally, it is important to note that ChannelsDB 2.0 is based on PDBe and AlphaFill structures availability, and PDBe and UniProt APIs. Hence, when any of these services have an outage, the visualisation of the results will fail. ChannelsDB currently does not use automatic updates for the entire database, so any recently added structure at PDBe or AlphaFill databases will likely not be added to ChannelsDB 2.0 yet. The current update cycle is set to 6-month period.

## Conclusions

ChannelsDB represents a unique resource for any analysis, which includes the transport of ligands and other small molecules within biomacromolecular structures. The updated ChannelsDB 2.0 represents a significant step forward in further channel analyses, which may facilitate future studies devoted to a deeper understanding of the biological roles and evolution of these functional features of biomacromolecular interactions with other molecules.

## Data Availability

The updated ChannelDB 2.0 website is available at https://channelsdb2.biodata.ceitec.cz with three times more protein entries (69776 on 1st September 2023) than the previous version, covered by two established software packages, CAVER https://loschmidt.chemi.muni.cz/caverweb and MOLE https://mole.upol.cz/.
